# Peanut allergy may be overdiagnosed in younger siblings of those with confirmed peanut allergy

**DOI:** 10.1186/1710-1492-10-S1-A7

**Published:** 2014-03-03

**Authors:** Elana Lavine, Reza Alizadehfar, Yuka Asai, Gregory Shand, Laurie Harada, Mary Allen, Moshe Ben Shoshan, Ann Clarke

**Affiliations:** 1 Humber River Regional Hospital, Toronto, ON, Canada; 2 Division of Pediatric Allergy and Clinical Immunology, Department of Pediatrics, McGill University Health Centre, Montreal, QC, Canada; 3 Division of Dermatology, Department of Medicine, McGill University Health Centre, Montreal, QC, Canada; 4 Division of Clinical Epidemiology, Department of Medicine, McGill University Health Centre, Montreal, QC, Canada; 5 Anaphylaxis Canada (AC), Toronto, ON, Canada; 6 Allergy/Asthma Information Association (AAIA), Toronto, ON, Canada; 7 Division of Clinical Immunology and Allergy, Department of Medicine, McGill University Health Center, Montreal, QC, Canada

## Background

Siblings of children with peanut allergy (PNA) are reported to have a higher prevalence of PNA than the general population. This prevalence may have both genetic and environmental influences, but may also reflect incorrect PNA diagnoses with less rigorous usage of confirmatory tests in siblings. The Peanut Allergy Registry (PAR), a Canadian database of individuals with PNA, was used to assess whether siblings born after the diagnosis of PNA in the index case (i.e., child with PNA) were more likely to have never been exposed to peanut (PN) and to be diagnosed without an appropriate clinical history or testing.

## Methods

A questionnaire was distributed to all PAR families with at least one child with confirmed PNA. Data were collected on siblings’ history of exposure or allergic reaction to PN, and on the results of any confirmatory tests. Univariate and multivariate logistic regressions were performed to examine the association between characteristics of the index case/ siblings and the following outcomes in siblings: i) complete PN avoidance and ii) parent-reported diagnosis of PNA without corroborating positive diagnostic tests. Predictors included sibling age and gender; index case age at PNA diagnosis, co-morbidities, and severity of allergic reaction; parental education level and marital status, province of residence, and sibship size. A hierarchical model was generated using the WinBUGS program to account for potential clustering effects within families.

## Results

Among 935 PAR families surveyed, 748 (80%) responded, representing 922 siblings, with median age 11.7 (IQR 7.4-16.3) years; 56.9% of siblings were younger than index cases. Eighty-three percent of siblings had been exposed to PN. Eighty (of 922) siblings were reported as having PNA (8.8%, CI 6.9,10.6). Of 80, 34 (42%) had no preceding allergic reaction to PN; in 5 of these 34, testing was either not performed or not supportive of the diagnosis. In multivariate analysis, siblings born after the diagnosis in the index PNA case were more likely (Odds Ratio (OR) 5.2, 95% CI 2.2, 12.9) to have completely avoided peanut. In univariate analysis, siblings born after the diagnosis in the index case were more likely to be diagnosed with PNA without a history or confirmatory testing (OR, 12.7 95% CI, 1.3, 120.7). This association was not significant in the multivariate analysis.

## Conclusions

Younger siblings of children with PNA may be at risk of being labeled with PNA without sufficient confirmatory testing. It is crucial to develop guidelines for both physicians and families that would prompt use of confirmatory tests to establish the diagnosis of PNA and advocate against unjustified PN avoidance.

